# Phenolic Content and Antioxidant Capacity of Synthetic Hexaploid Wheats

**DOI:** 10.3390/plants12122301

**Published:** 2023-06-13

**Authors:** Vladimir P. Shamanin, Zeynep H. Tekin-Cakmak, Salih Karasu, Inna V. Pototskaya, Sergey S. Shepelev, Alexandr S. Chursin, Alexey I. Morgounov, Osman Sagdic, Hamit Koksel

**Affiliations:** 1 Department of Agronomy, Breeding and Seed Production of the Agrotechnological Faculty, Omsk State Agrarian University, 1 Institutskaya Pl., 644008 Omsk, Russia; vp.shamanin@omgau.org (V.P.S.); iv.pototskaya@omgau.org (I.V.P.); ss.shepelev@omgau.org (S.S.S.);; 2 Department of Nutrition and Dietetics, Health Sciences Faculty, Istinye University, İstanbul 34010, Turkey; hazal.cakmak@yildiz.edu.tr; 3 Department of Food Engineering, Faculty of Chemical and Metallurgical Engineering, Davutpasa Campus, Yildiz Technical University, Istanbul 34349, Turkey; skarasu@yildiz.edu.tr (S.K.); osagdic@yildiz.edu.tr (O.S.); 4 Science Department, S. Seifullin Kazakh Agrotechnical University, Astana 010011, Kazakhstan

**Keywords:** synthetic hexaploid wheat, free phenolic, bound phenolic, gallic acid

## Abstract

In this study, 21 synthetic hexaploid wheat samples were analyzed and compared for phenolic content (the Folin–Ciocalteu method), phenolic compositions, and antioxidant activity (DPPH, ABTS, and CUPRAC). The aim of the study was to determine the phenolic content and antioxidant activity of synthetic wheat lines developed from *Ae. Tauschii*, which has a wide genetic diversity, to be used in breeding programs for developing new varieties with better nutritional properties. Bound, free, and total phenolic contents (TPCs) of wheat samples were determined as 145.38–258.55 mg GAE/100 g wheat, 188.19–369.38 mg GAE/100 g wheat, and 333.58–576.93 mg GAE/100 g wheat, respectively. Phenolic compositions were detected by the HPLC system. Gallic acid was found in the highest concentrations in free fractions, whereas gallic, p-coumaric acid, and chlorogenic acid were generally found in the highest concentrations in bound fractions of the synthetic hexaploid wheat samples. The antioxidant activities (AA%) of the wheat samples were evaluated by the DPPH assay. AA% in the free extracts of the synthetic red wheat samples ranged from 33.0% to 40.5%, and AA% values in the bound extracts of the synthetic hexaploid wheat samples varied between 34.4% and 50.6%. ABTS and CUPRAC analyses were also used to measure antioxidant activities. The ABTS values of the free and bound extracts and total ABTS values of the synthetic wheat samples ranged from 27.31 to 123.18, 61.65 to 263.23, and 93.94 to 308.07 mg TE/100 g, respectively. The corresponding CUPRAC values of the synthetic wheats were between 25.78–160.94, 75.35–308.13, and 107.51–364.79 mg TE/100 g. This study revealed that synthetic hexaploid wheat samples are valuable resources for breeding programs for developing new wheat varieties with higher concentrations and better compositions of health-beneficial phytochemicals. The samples w1 (Ukr.-Od. 1530.94/*Ae. squarrosa* (629)), w18 (Ukr.-Od. 1530.94/*Ae. squarrosa* (1027)), and w20 (Ukr.-Od. 1530.94/*Ae. squarrosa* (392)) can be used as a genetic resource in breeding programs to enhance the nutritional quality of wheat.

## 1. Introduction

Wheat (*Triticum aestivum*) is one of the most important foods for humans [1]. The majority of the wheat available on the market has white or red grains. Some unusual wheats, including red and blue wheat grains, are also commercially available, albeit in small quantities, thanks to their potential antioxidant properties and associated health benefits [2].

Tetraploid durum wheat (*Triticum turgidum*, AABB) and diploid wild goat grass (*Aegilops tauschii* Coss., DD) are intentionally hybridized to produce synthetic hexaploid wheat (SHW, AABBDD). In order to bridge the gene transfer from goat grass and durum wheat to hexaploid bread wheat, more than 1000 accessions have been generated at the International Maize and Wheat Improvement Centre (CIMMYT) until now. This is a rare instance for large-scale breeding success using wild relatives of wheat. The evolution of tetraploid and hexaploid wheat samples has been significantly influenced by the genus *Aegilops* L., which has considerable allelic diversity compared to wheat. It comprises 22 species with various genomes (C, D, M, N, S, T, and U) [3,4].

The re-synthesized wheat was produced by crossing modern durum wheat with *Ae. tauschii*, the likely source of the D-genome in hexaploid bread wheat. This has added novel genetic diversity to the wheat gene pool for a variety of traits [5,6]. Due to its high protein content (between 17.3 and 23.0%), *Ae. tauschii* can be utilized as a genetic resource [7]. The increased usage of synthetic hexaploid wheat has resulted in the introduction of new puroindoline alleles. In 92 breeding lines obtained from diverse crossings with synthetic wheat, the textural impacts of a number of Pina and Pinb alleles derived from *Ae. tauschii* were examined [8].

Evaluation of the 43 synthetic hexaploid wheat genotypes generated from crossing durum wheat ‘Langdon’ with 43 *Ae. tauschii* accessions revealed the presence of 11 1Dx and eight 1Dy subunits, including the recently discovered ones [9]. At the Glu-Dt1 gene, seven different allelic variations were found, three of which (1Dx1.5 + 1Dy10, 1Dx1.5 + 1Dy12.2, and 1Dx2.1 + 1Dy10) have not previously been identified in bread wheat germplasm [10]. Synthetic hexaploid wheats with the *Ae. tauschii* genome exhibit wide polymorphism in the loci controlling grain size, shape, and weight [11]. Although environmental conditions and genotype × environment interactions impact phenolic acid content, there is considerable genetic diversity in the phenolic components of wheat germplasm that might be utilized to produce new lines with greater phenolic acid levels [12].

Data on the phenolics content of wild wheat samples are rare and contradictory. This might be due to different analytical methods applied and the genotypic diversity of the wild wheats. Particularly, einkorn, emmer, and Khorasan wheat have higher contents and compositions of bioactive components than bread wheat and durum wheat [1,13]. According to Shelenga, Malyshev [14], it is possible to develop wheat and triticale cultivars with high resistance to fungal diseases (powdery mildew, leaf rust, and yellow rust) by using *Ae. tauschii* accessions with a high content of nonproteinogenic amino acids, polyols, phytosterols, acylglycerols, mono- and oligosaccharides, glycosides, and phenolic compounds.

The genetic diversity of *Ae. tauschii* is very rich on the south-western coast of the Caspian Sea (*Ae. tauschii* ssp. *tauschii*) in Azerbaijan and the northern regions of Iran (*Ae. tauschii* ssp. *strangulata*) [15]. Previous studies have shown that synthetic wheat lines with the *Ae. tauschii* genome are useful genetic resources for increasing the yield, resistance to diseases, and grain quality of spring-type bread wheat cultivars in Western Siberia [15]. 

This is a pioneering study to evaluate the phenolic compounds and antioxidant capacities of synthetic hexaploid wheat lines based on *Ae. tauschii* accessions from the “Caspian Basin region”. The purpose of this study is to identify sources of increased phenolic content and antioxidant activity among synthetic wheat lines based on the genetic diversity of *Ae. tauschii* for use in breeding programs as a genetic resource that could be used to develop new varieties to be utilized in wheat products with better nutritional properties.

## 2. Results and Discussions

### 2.1. The Free, Bound, and Total Phenolics

The free, bound, and total phenolics (sum of the free and bound fractions) values of synthetic wheat lines are presented in Table 1. The free, bound, and total phenolics values of the synthetic hexaploid wheat samples were in the range of 145.38–258.55, 188.19–369.38, and 333.57–576.94 mg GAE/100 g wheat, respectively. The total phenolic contents of wild goat grass and durum wheat (cv. Zenith) were determined as 146.22 and 198.58 mg GAE/100 g, respectively. Since these samples were not grown under the same environmental conditions, they are given here for a rough comparison and not included in the tables. There were significant (*p* < 0.05) differences between the total phenolic values of the synthetic wheat lines. Shelenga et al. [14] reported that synthetic hexaploid wheats CPI133872 and CPI133859 resistant to root-lesion nematodes revealed high levels of total phenolics in their root tissue, 576 µg and 518 µg GAE/g, respectively [16,17]. These variations in total phenolic values reported in the literature can be attributed to the various wheat types and extraction processes utilized [16,17]. After comparing with the literature results, it can be concluded that the total phenolics of synthetic hexaploid wheat samples analyzed in this study are quite high. This new synthetic hexaploid wheat can be considered as a new antioxidant-rich wheat genotype.

The samples w9 (333.57 mg GAE/100 g wheat) and w20 (576.94 mg GAE/100 g wheat) had the lowest and highest total phenolic values among the synthetic wheat genotypes (Table 1). In all of the synthetic wheat lines, the amount of bound phenolics was greater than the amount of free phenolics. Due to their strong antioxidant capacities and ability to prevent the oxidation of bioactive substances in the colon, the bound phenolic compounds serve significant functions [18]. The percentage of bound phenolics to the total phenolics was reported as 53–69% by Okarter et al. [19]. Liyana-Pathirana and Shahidi [18] also reported that the bound phenolic contributions of flours, whole grains, and brans in wheat samples were 40, 60, and 80%, respectively. Lacko-Bartošová, Lacko-Bartošová [20] reported that bound phenolic of the wheat was more than 80% among the total phenolic content. Among the synthetic wheat samples; W1 had the greatest free phenolic content, W13 had the highest bound phenolic content, and W20 had the highest total phenolic values in this investigation. W16, W17, W18, and W21 are sister lines. Although there were some significant differences in their TPC values (503.75, 516.68, 550.88, and 486.16 mg GAE/100 g, respectively), the variations among them were much lower than the rest of the samples. Furthermore, some of them had quite close TPC values (especially W16, W17, W21). The total phenolic values of W2, W4, and W15 were determined as 421.78, 455.86, and 411.16 mg GAE/100 g, respectively (Table 1).

The synthetic hexaploid wheats with the *Ae. tauschii* genome exhibit wide polymorphism in the loci controlling grain size, shape, and weight [11,21]. There is significant genetic diversity in the phenolic compounds of wheat germplasm, which could be utilized to select wheat genotypes with greater phenolic acid contents in grains. However, environmental variables and interactions between genotype and environment can also have an impact on the phenolic acid content [12]. According to Shelenga et al. [14], *Ae. tauschii* accessions with high concentrations of phenolics could be utilized to develop wheat and triticale cultivars that are highly resistant to the fungi that cause powdery mildew, brown rust, and yellow rust.

### 2.2. Individual Phenolic Contents

Phenolic acids provide several health benefits because of their strong antioxidant activity, which prevents oxidative cell damage by scavenging free radicals [22]. Regular consumption of phenolic acids also supports the anti-inflammation capacity. Wheat is frequently consumed by humans [23], and the development of wheats high in phenolic compounds has long been a priority. Dinelli et al. [24], and Sharma et al. [25] examined phenolic acids in bread and durum wheat and reported that phenolic acids are among the most significant phenolic compounds. Since wheat is widely consumed around the world, it can be considered as an important source of phenolic acids for the human diet.

Table 2 and Table 3 indicate, correspondingly, the phenolic compositions of free and bonded phenolic extracts of 21 distinct synthesized wheat samples. Depending on the wheat types, different phenolic acid patterns were observed, as shown in Table 2 and Table 3. In the free extracts of the synthetic wheats, gallic acid was the most common phenolic acid (Table 2). Gallic acid content in the free extracts of wheat samples were in the range of 11.20–92.73 mg/g of dry weight. The free fractions of the wheat samples, W21 and W6 had the lowest and the highest level of gallic acids, respectively. Protocatechuic acid (8.37–26.99 mg/g of dry weight), ellagic acid (8.03–35.04 mg/g of dry weight) and quercetin (16.73–21.84 mg/g of dry weight) were the other most abundant phenolic acids in the free fractions of the synthetic wheat samples. The phenolic acid composition of the wheat affected antioxidant activity because the phenolic acid showed different radical scavenging capacities. The free radical binding capacity of phenolic acids reduces as follows: gallic, caffeic, benzoic, sinapic, syringic, ferulic, p-coumaric, vanillic, and 4-hydroxybenzoic [20,26].

Table 3 also reveals that the main phenolic acids present in the bound fractions of the synthetic wheat samples were protocatechuic, gallic, and p-coumaric acids. The amount of protocatechuic acid in the bound fraction of the samples was between 0.00 and 85.55 mg/g of dry weight. The bound fractions of the samples, W3, W12, W17, and W13 exhibited a somewhat higher ferulic acid concentration (7.57, 8.67, 8.76, and 7.50 mg/g of dry weight, respectively). The most prevalent phenolic acid reported in whole grain wheat samples was ferulic acid, which is a derivative of hydroxycinnamic acid [27,28]. 

Ferulic acid content in the insoluble-bound fraction was between 87.4% and 97.2%, according to Okarter et al. [19]. and Liu et al. [2]. When they compared the phenolic acid contents of red, yellow, synthetic, and ordinary wheat, they found that synthetic wheat had the highest ferulic acid composition with a content of 81.38 mg/100 g. Lacko-Bartošová, Lacko-Bartošová [20] reported that Ferulic (66.3%) was the most prevalent free PA, followed by syringic (11.7%), sinapic (7.4%), p-hydroxybenzoic (5.3%), salicylic (3.8%), p-coumaric (3.6%), and caffeic (2.1%). The majority (94.0%) of all bound PAs were composed of bound ferulic acid, followed by p-coumaric acid (2.8%). Zhang, Wu [29] reported similar results related to bound and free phenolic composition. The findings revealed that the synthetic wheat genotypes utilized in the present study had a considerably greater amount of bound phenolic acids and that the distribution of bound phenolic acids was different from that of the free phenolic acids.

### 2.3. Antioxidant Activities with DPPH, ABTS and CUPRAC Assays

The antioxidant activity (AA%) values of the synthetic hexaploid wheat samples assessed by the DPPH method are given in Table 4. AA% values in the free and bound phenolic extracts of the synthetic hexaploid wheat samples were in the range of 33.0–40.5% and 34.4–50.6%, respectively. The samples, W1, W6, W18, and W20 had the highest AA% values in their bound phenolic extracts (50.6, 42.7, 43.5, and 42.6%, respectively) and they were also in the highest group in terms of total phenolics, except W6 (W1: 563.04 mg GAE/100 g, W18: 550.87 mg GAE/100 g, and W20: 576.93 mg GAE/100 g,). The findings revealed a positive correlation between the total phenolic values and the DPPH radical scavenging activities of the synthetic wheat samples. In repeated testing conducted over two years at seven different locations in China, the synthetic wheat cultivars Shumai-969 and Chuanmai-104 demonstrated their capacity to generate antioxidants [16,17]. This was consistent with the current study’s results.

There were significant differences in both free and bound AA% of the synthetic lines (*p* < 0.05). Furthermore, the free and bound phenolic extracts of the synthetic wheat samples behaved differently in terms of antioxidant activity. For example, the sample W1 had the highest bound AA% (50.6%), and its bound phenolic content was also in the highest group. On the other hand, the sample W17 had the greatest free AA% (40.5%) but its free phenolic content was not in the highest group. These results imply that the free and bound antioxidant capacity is affected by the genotype in synthetic wheat, and wheat varieties with high-antioxidant activity could be developed by taking these factors into consideration. The antioxidant activity has a strong relationship with total phenolic content and phenolic composition [20]. The samples with higher phenolic and Gallic acid contents showed a higher DPPH value. 

According to the study’s findings, bound phenolic compounds have higher DPPH radical scavenging potential than free phenolic compounds. These results are in line with the antioxidant activity results of the samples, which showed that the antioxidant activities of bound phenolics were greater than those of free phenolics in all fractions. Di Loreto et al. [30] reported that the highest antioxidant activity value (DPPH) was found in the old wheat cultivar, Inglesa (7.44 µmol TE/g), whereas the lowest antioxidant activity value was obtained in the modern durum wheat variety, Claudio (4.06 µmol TE/g). According to another study, the antioxidant and chelating activity of wheat varies depend on both its genotype and the grooving environment [30,31,32]. 

ABTS and CUPRAC analyses were also used to measure antioxidant activities. Due to its simplicity of use and the stability of the ABTS radical, the ABTS analysis has been utilized quite frequently to assess the antioxidant potential of food, biological materials, and pure substances. Antioxidants participate in the CUPRAC analysis by reacting by giving an electron, turning Cu^2+^ into Cu^+^. Table 5 provides the ABTS values for the samples of synthesized hexaploid wheat that contains free, bound, and total ABTS (sum of the free and bound fractions) preparations. The ABTS values of the synthesized hexaploid wheat samples’ free and bound extracts and total ABTS values ranged from 27.31 to 123.18 mg TE/100 g, from 61.65 to 263.23 mg TE/100 g, and from 93.94 to 308.07 mg TE/100 g, respectively. The ABTS values of the seven wheat samples that ranged from 51.13 to 92.30 mg TE/100 g wheat kernels were reported by Zengin, Nithiyanantham [33]. 

The CUPRAC values of the free and bound extracts and total CUPRAC (sum of the free and bound fractions) of synthetic hexaploid wheat samples are reported in Table 6. The CUPRAC values of the free and bound extracts and total CUPRAC values of synthetic hexaploid wheat samples were between 25.78 and 160.94 mg TE/100 g, 75.35 and 308.13 mg TE/100 g, and 107.51 and 364.79 mg TE/100 g, respectively. Zengin, Nithiyanantham [33] also reported that CUPRAC reduced the power values of the seven wheat cultivar samples to between 116.03 mg TE/100 g wheat grains and 242.47 mg TE/100 g wheat grains.

## 3. Materials and Methods

### 3.1. Raw Material

The phenotyping of synthetic hexaploid lines (SHL) was conducted at Omsk State Agrarian University (55°02′ N, 73°32′ E; Omsk, Russia) in 2021. In 2016–2020, 126 spring and winter wheat synthetic lines were evaluated and identified previously [15], from which the best 21 SHW lines (Table 7) adapted to local climate conditions were evaluated in 2021. These SHWs included CIMMYT synthetic hexaploid wheat lines, which were developed by crossing goat grass (*Ae. tauschii* Coss., D genome, syn. *Ae. squarrosa*, *Ae. sq.*) from the western coast of Caspian Sea (Iran and Azerbaijan) with durum wheat cultivars from the Institute of Breeding and Genetics (Odessa, Ukraine) and the cultivar Pandur from Romania (*T. durum Desf*., AB). 

**Figure 1:**
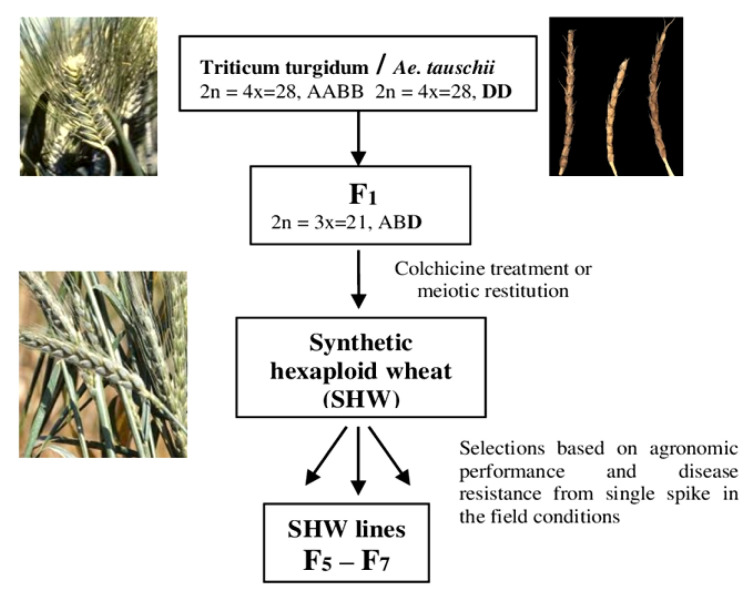
Breeding scheme for development of synthetic hexaploid wheat lines based on the elite durum wheat varieties and accession of *Ae. tauschii*.

Figure 1 indicates that the lines were chosen from single spike selections in F5–F7 of each hybrid combination, according to the agronomic performance and disease resistance. Kyoto University (Japan) developed one line through crosses of Langdon durum (*T. turgidum* L.) and the accession of *Ae. tauschii* IG 12638. In each hybrid combination, some promising breeding lines were selected during evaluation in the field conditions, which were transferred to Omsk SAU for further evaluation.

All synthetic hexaploid lines are shown in Table 8 and W2-4-15, W5-6-20, W7-16-17-18-21, W8-9-19, W11-14 are the sister lines of each other, respectively. The grains were ground by using a laboratory grinder (CemotecTM, CM290, Hillerod, Denmark) for 90 s.

### 3.2. Chemicals

The following chemicals were acquired from Sigma-Aldrich: hexane, acetone, diethyl ether, ethyl acetate, Folin–Ciocalteu reagent, and 1,1-diphenyl-2-picryl-hydrazil (DPPH) (Bornem, Belgium). ICN Biomedicals, Inc. supplied the gallic acid (Aurora, OH, USA). Merck supplied analytical grade methanol, copper (II) chloride, absolute ethyl alcohol, ammonium acetate, and glacial acetic acid (Darmstadt, Germany).

### 3.3. Extraction

#### 3.3.1. Removal of Oil from Wheat Samples

The ground samples were defatted prior to analysis. Hexane was added to the wheat sample at a ratio of 1 g:5 mL, *w*/*v* and mixed by a vortex. The samples were shaken at 200 rpm for 10 min by a shaker (MK200D, Yamato Scientific Co., Ltd., Tokyo, Japan). Then, centrifuged (Heraeus, Multifuge X3 FR, Thermo Scientific, Dreieich, Germany) for 5 min at 2500× *g*. The defatting procedure was repeated three times, and the samples were left for drying (12 h) in a fume hood.

#### 3.3.2. Extraction of Free Phenolic Compounds

The extraction of free phenolics from wheat samples was performed as described previously [34]. The defatted sample was mixed with a vortex after adding a solution of acetone and water (1:1). The extraction was repeated three times. The supernatants were collected in a tube wrapped with aluminum foil, and kept at +4 °C. The precipitates were dried overnight at 30 °C. The solvent in the supernatant was evaporated (Hei-VAP Advantage, Heidolph, Germany) as described previously [34]. The dry phenolic compounds were dissolved in methanol (4 mL) in the evaporation flask wrapped with aluminum foil. It was shaken for 15 min at one-minute intervals. The dissolved samples were placed into 4 mL amber-colored vials and kept at −18 °C.

#### 3.3.3. Extraction of Bound Phenolic Compounds

The extraction of bound phenolics from wheat samples was performed as described previously [34]. The residual pellet was hydrolyzed with 2N NaOH (20 mL) for 4 h. The pH was arranged to 2.0 ± 0.2 by adding 6M HCl. The extraction was performed five times to remove residual free fatty acids. In each extraction, 10 mL of hexane was added, vortexed, shaken for 10 min at 200 rpm and centrifuged (Heraeus, Multifuge X3 FR, Thermo Scientific, Germany) for 10 min at 4000× *g*. The supernatant (hexane + free fatty acids) was removed. Then, the extraction of bound phenolics was carried out with 10 mL of diethyl ether-ethyl acetate (1:1, *v*/*v*). Six extractions were performed, and all of the diethyl ether-ethyl acetate fractions were combined. Then, the solvents were evaporated (Hei-VAP Advantage, Heidolph, Germany), as described previously [34]. The dry phenolic compounds were dissolved in 2 mL of methanol in the evaporation flask wrapped with aluminum foil. It was shaken for 15 min at one-minute intervals. The dissolved samples were placed into 4 mL amber-colored vials and kept at −18 °C.

### 3.4. Free, Bound, and Total Phenolic Contents

The Folin–Ciocalteu method was modified to detect the concentrations of free and bound phenolic compounds, and their sum was used to estimate the total phenolics. To summarize, 2N Folin–Ciocalteu reagent (500 µL), 200 g/L Na_2_CO_3_ solution (1.5 mL), and distilled water (7.9 mL) were combined with 100 µL of methanol extract and kept in the dark for 120 min. Then, it was centrifuged for 5 min at 4000× *g*, and the absorbance was determined at 760 nm by a spectrophotometer (Shimadzu 150 UV-1800, Kyoto, Japan). The phenolic contents were reported as gallic acid equivalents (GAE). 

### 3.5. HPLC Determination of Individual Phenolics

The analysis of phenolic acid profiles of the extracts was performed with some modifications, as described previously [35]. The extracts were filtered by using a filter (0.22 µm). An Agilent 1200 HPLC system composed of a photodiode array detector (HLPC-DAD), quaternary pump, autosampler, column oven (Shimadzu Corp., Kyoto, Japan), and Waters Atlantis C18 column (250 mm × 4.6 mm, 5 m) was used for the chromatographic analyses. A linear gradient elution procedure with solvents A and B in the ratios of 0.1:99.9, *v*/*v* (acetic acid/water and acetonitrile, respectively) was carried out and used to separate phenolic acids (gallic acid, protocatechuic acid, catechin, syringic acid, ellagic acid, m-coumaric acid, o-coumaric acid, chrysin, cafeic acid, p-coumaric acid, ferulic acid, myricetin, quercetin, kaempferol, rutin, sinapic acid, and chlorogenic acid) on the C18 column. The flow rate of the solvents was 1 mL/min. The solvent gradient was programmed as follows: linear-gradient elution from 10% B to 60% B, 0–15 min; isocratic elution of 60% B, 15–20 min; linear gradient elution from 60% B to 10% B, 20–25 min; and isocratic elution of 10% B, 25–30 min. The chromatograms were recorded at 278 nm, 320 nm, and 360 nm by monitoring spectra within the wavelength range 190–400 nm. Identification of phenolic acids was accomplished by comparing the retention time and absorption spectra of peaks in wheat samples to those of standard compounds. The quantitation of phenolic acids was based on calibration curves built for each of the compounds identified in the samples.

### 3.6. DPPH Radical Scavenging Activity

The antioxidant capacity was performed as described by Singh et al. [36] with the DPPH radical scavenging activity method. In this method, 4.9 mL of fresh 1,1-diphenyl-2-picrylhydrazil (DPPH) solution was added to 100 µL of the wheat extract. The absorbance value of the solution was measured at 515 nm by a Shimadzu 150 UV-1800 spectrophotometer, following incubation at 30 °C for 60 min (Kyoto, Japan). The results are reported as mg TE/100 g ground wheat sample.

### 3.7. ABTS Scavenging Activity

The ABTS radical-cation scavenging capacity of the wheat extracts was performed with some modifications, with the method described by Rice-Evans and Miller [37]. First, 2 mL of ABTS solution was added to 100 µL of extract and incubated at 30 °C for 6 min. After incubation, the absorbance of the solution was measured at 734 nm by a spectrophotometer (Shimadzu 150 UV-1800, Kyoto, Japan). The results are expressed as mg TE/100 g wheat. 

### 3.8. CUPRAC (CUPric Reducing Antioxidant Capacity) Assay

Copper (II) chloride solution (10^−2^ M) was prepared from CuCl_2_·2H_2_O, dissolved in H_2_O. By dispersing ammonium acetate (NH_4_Ac) in water, a buffer solution with a pH of 7.0 was prepared. Neocuproine (Nc) was dissolved in 96% EtOH and then diluted to 25 mL with ethanol to make a solution with a concentration of 7.5 × 10^−3^ M. Then, 1 mL of CuCl_2_ (A), 1 mL of Neocuproine (B), 1 mL of NH_4_Ac (C), 0.1 mL of sample, and 1 mL of water were added to the extract, respectively. The solution was vortexed for 20 s, and absorbance measurement was performed exactly after 60 min at 450 nm by a spectrophotometer (Shimadzu 150 UV-1800, Kyoto, Japan) [38].

### 3.9. Statistical Analysis

The analytical results are presented as the mean and standard deviation of at least two separate extractions. The significance of mean differences was assessed using Tukey’s post hoc test following the application of ANOVA to the findings in SPSS version 9.0 (SPSS Inc., Chicago, IL, USA).

## 4. Conclusions

TPC, phenolic compositions, and the antioxidant activities of the synthetic hexaploid wheat samples based on *Ae. tauschii* accessions from the “Caspian Basin region” were measured. Significant differences were detected between the phenolic contents/composition as well as the AA% values of the synthetic wheat samples. Gallic acid was the predominant phenolic acid found in the free fractions of the synthetic wheat samples, while gallic and p-coumaric acids were the predominant phenolic acids in the bound fractions. The significant differences between the antioxidant activities of the synthetic lines indicate that synthetic wheat genotypes have great variability in terms of antioxidant capacity, which should be taken into consideration in the development of high-antioxidant wheats with health benefits. It can be concluded that selected synthetic wheat lines (W1, W6, W18, and W20) with the highest bound AA% could be used in breeding programs to develop new bread wheat varieties with a high antioxidant capacity suitable for the production of functional food.

## Figures and Tables

**Table 1:** Free, bound, and total phenolics of synthetic hexaploid wheat samples.

Sample	Free Phenolics (mg GAE/100 g)	Bound Phenolics (mg GAE/100 g)	Total Phenolics (mg GAE/100 g)
W1	258.55 ± 0.1 ^a^	304.49 ± 0.6 ^f,g^	563.04 ± 0.7 ^a,b^
W2	180.78 ± 1.8 ^h,i^	241.00 ± 0.5 ^l^	421.78 ± 2.3 ^h,i^
W3	184.17 ± 0.5 ^g,h,i^	228.03 ± 0.8 ^n^	412.20 ± 1.3 ^h,i,j^
W4	198.58 ± 0.3 ^d,e,f^	257.28 ± 0.2 ^k^	455.86 ± 0.5 ^f^
W5	198.18 ± 0.1 ^d,e,f^	238.84 ± 0.2 ^l^	437.01 ± 0.3 ^g^
W6	183.74 ± 0.7 ^g,h,i^	227.61 ± 0.4 ^n^	411.35 ± 1.1 ^i,j^
W7	197.31 ± 0.2 ^d,e,f,g^	201.33 ± 0.3 ^p^	398.64 ± 0.5 ^j^
W8	187.13 ± 0.3 ^f,g,h,i^	213.20 ± 0.8 ^o^	400.28 ± 1.3 ^j^
W9	145.38 ± 0.1 ^j^	188.19 ± 0.1 ^q^	333.57 ± 0.2 ^k^
W10	240.32 ± 0.1 ^b,c^	299.24 ± 0.1 ^h^	539.56 ± 0.2 ^c^
W11	193.49 ± 0.3 ^e,f,g,h^	356.03 ± 0.9 ^c^	549.51 ± 1.2 ^b,c^
W12	205.99 ± 0.9 ^d,e^	361.40 ± 0.7 ^b^	567.39 ± 1.6 ^a^
W13	199.21 ± 0.9 ^d,e,f^	369.38 ± 0.6 ^a^	568.59 ± 1.5 ^a^
W14	192.22 ± 0.5 ^f,g,h^	234.53 ± 0.9 ^m^	426.75 ± 1.4 ^g,h^
W15	188.62 ± 0.0 ^i^	233.54 ± 1.0 ^m^	411.16 ± 1.0 ^i,j^
W16	229.09 ± 0.1 ^c^	274.66 ± 0.3 ^j^	503.75 ± 0.3 ^d^
W17	210.66 ± 0.3 ^d^	306.02 ± 0.0 ^f^	516.68 ± 0.3 ^d^
W18	248.38 ± 0.2 ^a,b^	302.49 ± 0.5 ^g^	550.88 ± 0.7 ^b,c^
W19	193.49 ± 0.0 ^e,f,g,h^	320.85 ± 0.2 ^e^	514.34 ± 0.2 ^d^
W20	231.64 ± 0.5 ^c^	345.30 ± 0.1 ^d^	576.94 ± 0.6 ^a^
W21	200.48 ± 0.2 ^d,e,f^	285.68 ± 0.4 ^i^	486.16 ± 0.6 ^e^

Means with different letters in each column are significantly different.

**Table 2:** Free phenolic compounds of synthetic hexaploid wheat samples (mg/g of dry weight).

	Gallic Acid	Protocatechuic Acid	Catechin	Syringic Acid	Ellagic Acid	Chrysin	Caffeic Acid	p-Coumaric Acid	Ferulic Acid	Quercetin	Kaempferol	Chlorogenic Acid	Rutin	Sinapic Acid
W1	28.69	18.31	4.85	1.46	12.24	1.20	4.81	2.03	7.09	17.14	6.51	2.19	9.95	6.07
W2	21.10	15.84	4.67	0.29	8.87	0.72	4.32	2.13	6.07	16.97	6.71	1.18	10.56	4.99
W3	25.77	14.41	4.06	0.44	16.51	0.60	4.23	1.67	5.07	16.81	6.43	1.22	7.85	3.92
W4	29.46	17.35	6.19	0.26	19.16	0.66	4.45	2.46	6.40	17.23	7.95	1.34	12.47	5.34
W5	87.35	13.56	7.22	0.28	13.16	1.00	4.29	1.97	5.89	16.87	6.68	1.42	9.64	4.80
W6	92.73	20.32	0.00	1.37	9.60	1.09	4.27	1.07	6.20	18.16	6.69	1.15	4.43	5.13
W7	45.11	26.48	5.68	0.32	10.54	3.19	6.21	3.67	13.10	17.60	7.58	2.78	19.47	12.49
W8	39.39	13.23	2.77	0.82	8.06	0.00	4.90	0.92	6.22	16.83	6.48	1.04	3.56	5.15
W9	41.23	12.50	3.38	0.73	18.65	15.92	5.14	1.94	6.03	16.81	6.53	0.91	9.43	4.95
W10	14.54	15.02	3.25	0.45	11.18	2.17	4.43	1.73	7.81	17.08	6.50	1.51	8.22	6.84
W11	49.51	11.79	9.64	1.23	12.33	4.63	4.38	1.50	4.14	17.07	6.52	1.19	6.87	2.94
W12	46.90	26.99	3.70	0.72	26.07	4.38	4.30	4.33	8.67	16.73	6.50	2.45	23.25	7.76
W13	20.93	11.68	3.58	0.19	17.08	3.16	4.54	1.12	5.92	17.29	6.47	1.27	4.69	4.83
W14	41.25	17.19	0.00	1.10	11.53	4.13	4.45	0.75	7.28	19.63	6.40	1.97	2.54	6.28
W15	24.07	22.81	5.38	2.40	11.74	3.36	4.74	1.30	8.13	18.72	6.48	1.19	5.74	7.18
W16	30.44	19.12	4.38	2.38	12.79	3.93	5.04	2.43	9.93	21.84	6.70	1.50	12.28	9.10
W17	32.02	15.19	3.41	0.70	10.11	3.70	4.75	1.48	8.76	16.87	6.68	2.36	6.76	7.86
W18	28.57	17.22	0.00	0.09	8.03	0.95	5.15	1.47	5.53	17.12	6.66	0.86	6.73	4.42
W19	33.08	15.91	0.00	0.91	35.04	0.00	4.84	1.63	11.74	16.83	6.66	1.08	7.63	11.04
W20	30.38	8.37	7.03	1.41	8.67	1.03	4.83	3.19	10.74	16.90	6.50	0.81	16.68	9.97
W21	11.20	16.66	0.00	1.98	9.58	0.16	4.42	1.22	5.76	16.72	6.50	0.96	5.28	4.66

**Table 3:** Bound phenolic compounds of synthetic hexaploid wheat samples (mg/g of dry weight).

	Gallic Acid	Protocatechuic Acid	Catechin	Syringic Acid	Ellagic Acid	Chrysin	Caffeic Acid	p-Coumaric Acid	Ferulic Acid	Quercetin	Kaempferol	Chlorogenic Acid	Rutin	Sinapic Acid
W1	14.98	3.28	17.59	0.00	0.00	0.36	4.30	16.74	6.34	0.28	3.10	14.98	3.28	17.59
W2	8.94	0.00	27.06	0.69	0.00	0.77	6.72	0.00	0.00	2.66	5.69	8.94	0.00	27.06
W3	8.32	0.00	9.50	1.11	4.12	12.69	39.77	16.68	7.57	10.59	32.51	8.32	0.00	9.50
W4	15.10	58.97	21.87	3.02	0.00	0.62	5.46	16.71	6.47	1.80	4.34	15.10	58.97	21.87
W5	9.25	0.00	41.93	1.96	0.00	1.19	13.13	17.06	6.33	5.13	12.52	9.25	0.00	41.93
W6	7.97	0.00	22.90	2.65	0.00	0.50	5.55	16.70	6.40	1.11	4.43	7.97	0.00	22.90
W7	17.16	51.30	0.00	0.62	0.00	0.38	1.91	0.00	6.70	0.42	0.56	17.16	51.30	0.00
W8	11.36	85.55	0.00	0.65	0.00	0.38	1.86	0.00	6.61	0.41	0.50	11.36	85.55	0.00
W9	8.37	0.00	33.59	0.00	4.27	3.55	12.85	16.89	6.58	18.76	18.82	8.37	0.00	33.59
W10	9.07	0.00	8.67	2.58	0.00	0.50	4.21	16.68	6.47	1.13	3.01	9.07	0.00	8.67
W11	8.15	31.54	17.66	3.10	0.00	0.31	2.88	0.00	6.55	0.01	1.58	8.15	31.54	17.66
W12	9.84	0.00	41.70	1.46	4.18	1.65	32.29	17.02	6.41	7.75	32.94	9.84	0.00	41.70
W13	15.92	19.13	9.78	0.00	4.22	1.37	24.74	19.51	7.50	6.17	24.89	15.92	19.13	9.78
W14	13.11	0.00	0.00	0.48	0.00	0.32	1.89	17.21	0.00	0.09	0.53	13.11	0.00	0.00
W15	9.62	0.00	9.74	1.58	0.00	0.37	2.01	16.71	0.00	0.35	0.66	9.62	0.00	9.74
W16	8.23	10.12	11.13	0.67	0.00	0.35	2.00	0.00	0.00	0.27	0.65	8.23	10.12	11.13
W17	11.62	5.04	12.07	0.66	4.17	1.17	26.41	17.98	7.15	5.01	26.67	11.62	5.04	12.07
W18	14.99	75.28	10.78	0.85	0.00	0.38	2.63	16.72	6.36	0.39	1.32	14.99	75.28	10.78
W19	12.62	0.00	9.91	2.27	4.16	0.68	10.03	17.01	6.61	2.15	9.21	12.62	0.00	9.91
W20	8.33	0.00	17.35	2.72	0.00	0.40	1.96	0.00	0.00	0.52	0.61	8.33	0.00	17.35
W21	9.52	7.37	10.53	0.30	0.00	0.35	1.88	16.72	0.00	0.24	0.53	9.52	7.37	10.53

**Table 4:** Antioxidant activity (DPPH) of synthetic hexaploid wheat samples.

# of Sample	Free (%AA)	Bound (%AA)
W1	36.5 ± 0.1 ^b^	50.6 ± 0.2 ^a^
W2	33.8 ± 0.0 ^h^	35.7 ± 0.1 ^h,i^
W3	34.0 ± 0.2 ^f,g,h^	41.8 ± 0.0 ^c^
W4	34.4 ± 0.0 ^e,f,g,h^	35.8 ± 0.0 ^h,i^
W5	33.8 ± 0.1 ^h,i^	36.1 ± 0.2 ^f,g,h^
W6	35.1 ± 0.0 ^d,e^	42.7 ± 0.0 ^b,c^
W7	35.1 ± 0.1 ^d,e,f^	36.7 ± 0.2 ^f,g^
W8	33.0 ± 0.0 ^i^	35.9 ± 0.0 ^g,h^
W9	34.0 ± 0.1 ^g,h^	35.0 ± 0.2 ^i,j^
W10	33.8 ± 0.1 ^h^	39.4 ± 0.3 ^e^
W11	33.3 ± 0.0 ^h,i^	40.6 ± 0.0 ^d^
W12	35.4 ± 0.0 ^c,d^	36.4 ± 0.0 ^f,g,h^
W13	33.8 ± 0.1 ^h,i^	39.9 ± 0.1 ^d,e^
W14	34.7 ± 0.0 ^d,e,f,g^	34.4 ± 0.0 ^j^
W15	35.4 ± 0.1 ^c,d^	39.2 ± 0.1 ^e^
W16	35.9 ± 0.0 ^b,c^	35.5 ± 0.2 ^h,i^
W17	40.5 ± 0.1 ^a^	40.7 ± 0.0 ^d^
W18	40.3 ± 0.0 ^a^	43.5 ± 0.1 ^b^
W19	35.9 ± 0.4 ^b,c^	36.9 ± 0.0 ^f^
W20	34.2 ± 0.0 ^f,g,h^	42.6 ± 0.4 ^c^
W21	34.3 ± 0.1 ^e,f,g,h^	36.2 ± 0.0 ^f,g,h^

Means with different letters in each column are significantly different.

**Table 5:** Antioxidant activity (ABTS) of synthetic hexaploid wheat samples.

# of Sample	Free (mg TE/100 g)	Bound (mg TE/100 g)	Total (mg TE/100 g)
W1	37.93 ± 0.03 ^k^	93.84 ± 0.04 ^n^	131.77 ± 0.07 ^n^
W2	31.13 ± 0.01 ^q^	62.82 ± 0.04 ^s^	93.94 ± 0.05 ^u^
W3	73.96 ± 0.02 ^c^	112.91 ± 0.01 ^l^	186.87 ± 0.03 ^k^
W4	123.18 ± 0.02 ^a^	166.19 ± 0.01 ^i^	289.37 ± 0.03 ^d^
W5	34.39 ± 0.00 ^n^	71.12 ± 0.05 ^r^	105.50 ± 0.05 ^s^
W6	45.52 ± 0.02 ^h^	76.76 ± 0.02 ^q^	122.27 ± 0.04 ^o^
W7	61.25 ± 0.01 ^e^	101.49 ± 0.07 ^m^	162.74 ± 0.08 ^m^
W8	40.63 ± 0.02 ^j^	77.07 ± 0.02 ^p^	117.70 ± 0.04 ^p^
W9	66.42 ± 0.04 ^d^	241.64 ± 0.04 ^b^	308.07 ± 0.08 ^a^
W10	27.31 ± 0.01 ^t^	184.09 ± 0.06 ^g^	211.39 ± 0.07 ^j^
W11	32.64 ± 0.01 ^o^	144.07 ± 0.09 ^k^	176.71 ± 0.10 ^l^
W12	43.66 ± 0.00 ^i^	224.56 ± 0.02 ^d^	268.22 ± 0.02 ^f^
W13	66.48 ± 0.02 ^d^	157.11 ± 0.07 ^j^	223.59 ± 0.09 ^i^
W14	51.92 ± 0.03 ^g^	198.03 ± 0.02 ^f^	249.95 ± 0.05 ^g^
W15	37.09 ± 0.01 ^l^	238.50 ± 0.05 ^c^	275.58 ± 0.06 ^e^
W16	29.67 ± 0.02 ^r^	263.23 ± 0.02 ^a^	292.89 ± 0.04 ^c^
W17	114.19 ± 0.03 ^b^	179.59 ± 0.04 ^h^	293.78 ± 0.07 ^b^
W18	36.07 ± 0.01 ^m^	202.52 ± 0.08 ^e^	238.60 ± 0.09 ^h^
W19	52.15 ± 0.02 ^f^	61.65 ± 0.01 ^t^	113.80 ± 0.03 ^q^
W20	32.08 ± 0.01 ^p^	62.88 ± 0.02 ^s^	94.97 ± 0.03 ^t^
W21	27.87 ± 0.00 ^s^	82.99 ± 0.03 ^o^	110.85 ± 0.03 ^r^

Means with different letters in each column are significantly different.

**Table 6:** Antioxidant activity (CUPRAC) of synthetic hexaploid wheat samples.

# of Sample	Free (mg TE/100 g)	Bound (mg TE/100 g)	Total (mg TE/100 g)
W1	90.21 ± 0.06 ^h^	128.13 ± 0.11 ^i^	218.34 ± 0.17 ^g^
W2	25.78 ± 0.05 ^r^	81.73 ± 0.01 ^s^	107.51 ± 0.06 ^q^
W3	58.53 ± 0.00 ^l^	155.62 ± 0.06 ^f^	214.15 ± 0.11 ^i^
W4	114.27 ± 0.01 ^e^	122.53 ± 0.20 ^k^	236.80 ± 0.21 ^f^
W5	43.04 ± 0.02 ^o^	75.35 ± 0.10 ^t^	118.39 ± 0.12 ^p^
W6	43.87 ± 0.01 ^o^	88.99 ± 0.08 ^q^	132.86 ± 0.09 ^o^
W7	127.03 ± 0.05 ^d^	146.55 ± 0.10 ^h^	273.56 ± 0.15 ^e^
W8	51.33 ± 0.15 ^n^	97.45 ± 0.11 ^o^	148.78 ± 0.26 ^m^
W9	89.73 ± 0.09 ^h^	112.93 ± 0.05 ^m^	202.66 ± 0.14 ^j^
W10	35.60 ± 0.14 ^q^	82.53 ± 0.05 ^r^	118.13 ± 0.19 ^p^
W11	61.73 ± 0.21 ^k^	113.41 ± 0.08 ^m^	175.14 ± 0.29 ^l^
W12	91.86 ± 0.19 ^g^	125.45 ± 0.07 ^j^	217.31 ± 0.26 ^h^
W13	77.63 ± 0.06 ^j^	158.55 ± 0.05 ^e^	236.18 ± 0.11 ^f^
W14	56.54 ± 0.15 ^m^	120.82 ± 0.22 ^l^	177.36 ± 0.37 ^k^
W15	131.30 ± 0.11 ^c^	147.62 ± 0.04 ^g^	278.95 ± 0.15 ^d^
W16	110.00 ± 0.33 ^f^	163.84 ± 0.15 ^d^	273.84 ± 0.48 ^e^
W17	160.94 ± 0.04 ^a^	203.86 ± 0.20 ^b^	364.79 ± 0.24 ^a^
W18	52.13 ± 0.10 ^n^	308.13 ± 0.03 ^a^	360.26 ± 0.13 ^b^
W19	39.52 ± 0.40 ^p^	107.80 ± 0.03 ^n^	147.13 ± 0.43 ^n^
W20	134.53 ± 0.10 ^b^	187.88 ± 0.15 ^c^	322.41 ± 0.26 ^c^
W21	80.67 ± 0.12 ^i^	96.41 ± 0.17 ^p^	177.08 ± 0.29 ^k^

Means with different letters in each column are significantly different.

**Table 7:** Pedigree of synthetic lines and origin of *Ae. tauschii* used in the synthetics.

Pedigree	Number of Lines	Cross ID	*Ae. tauschii* Origin	Subspecies
CIMMYT spring synthetics *				
Aisberg/*Ae.sq.*(369)	2	C04GH3	Mazandaran, Iran	*tauschii*
Aisberg/*Ae.sq.*(511)	3	C04GH5	Unknown	Unknown
Ukr.-od.952.92/*Ae.sq.*(1031)	3	C04GH61	Zanjan, Iran	*tauschii*
Ukr.-od.1530.94/*Ae.sq.*(310)	1	C04GH68	Gilan, Iran	*strangulata*
Ukr.-od.1530.94/*Ae.sq.*(392)	3	C04GH71	Shemakha, Azerbaijan	*tauschii*
Ukr.-od.1530.94/*Ae.sq.*(458)	1	C04GH74	Unknown	Unknown
Ukr.-od.1530.94/*Ae.sq.*(629)	1	C04GH76	Mazandaran, Iran	*strangulata*
Ukr.-od.1530.94/*Ae.sq.*(1027)	5	C04GH78	Mazandaran, Iran	*tauschii*
Pandur/*Ae.sq.*(223)	1	C04GH79	Gilan, Iran	*tauschii*
Japanese synthetics **				
Langdon/*Ae.sq.* IG 126387	1	–	Ashkhabad, Turkmenistan	Unknown

*—accessions of *Ae. tauschii* from CIMMYT Germplasm Bank. **—IG: International Center for Agricultural Research in the Dry Areas (ICARDA).

**Table 8:** Synthetic hexaploid wheat samples used in this study.

# of Samples	Pedigree
W1	Ukr.-Od. 1530.94/*Ae.squarrosa* (629)
W2	Ukr.-Od. 952.92/*Ae.squarrosa* (1031)
W3	Ukr.-Od. 1530.94/*Ae.squarrosa* (458)
W4	Ukr.-Od. 952.92/*Ae.squarrosa* (1031)
W5	Ukr.-Od. 1530.94/*Ae.squarrosa* (392)
W6	Ukr.-Od. 1530.94/*Ae.squarrosa* (392)
W7	Ukr.-Od. 1530.94/*Ae.squarrosa* (1027)
W8	Aisberg/*Ae.squarrosa* (511)
W9	Aisberg/*Ae.squarrosa* (511)
W10	Pandur/*Ae.squarrosa* (223)
W11	Aisberg/*Ae.squarrosa* (369)
W12	Langdon/IG 126387
W13	Ukr.-Od. 1530.94/*Ae.squarrosa* (310)
W14	Aisberg/*Ae.squarrosa* (369)
W15	Ukr.-Od. 952.92/*Ae.squarrosa* (1031)
W16	Ukr.-Od. 1530.94/*Ae.squarrosa* (1027)
W17	Ukr.-Od. 1530.94/*Ae.squarrosa* (1027)
W18	Ukr.-Od. 1530.94/*Ae.squarrosa* (1027)
W19	Aisberg/*Ae.squarrosa* (511)
W20	Ukr.-Od. 1530.94/*Ae.squarrosa* (392)
W21	Ukr.-Od. 1530.94/*Ae.squarrosa* (1027)

## Data Availability

The data presented in this study are available on request from the corresponding author.
